# Risk factors and outcomes of acute kidney injury in South African critically ill adults: a prospective cohort study

**DOI:** 10.1186/s12882-019-1620-7

**Published:** 2019-12-10

**Authors:** Ryan E. Aylward, Elizabeth van der Merwe, Sisa Pazi, Minette van Niekerk, Jason Ensor, Debbie Baker, Robert J. Freercks

**Affiliations:** 1 Adult Critical Care Unit, Livingstone Hospital, Port Elizabeth, South Africa; 2 Walter Sisulu University, Mthatha, South Africa; 3 Department of Statistics, Nelson Mandela University, Port Elizabeth, South Africa; 4 Division of Nephrology and Hypertension, Livingstone Hospital, Port Elizabeth, South Africa; 5 Department of Medicine, Division Nephrology and Hypertension, University of Cape Town, Cape Town, South Africa

**Keywords:** AKI, Africa, HIV, ICU, Dialysis

## Abstract

**Background:**

There is a marked paucity of data concerning AKI in Sub-Saharan Africa, where there is a substantial burden of trauma and HIV.

**Methods:**

Prospective data was collected on all patients admitted to a multi-disciplinary ICU in South Africa during 2017. Development of AKI (before or during ICU admission) was recorded and renal recovery 90 days after ICU discharge was determined.

**Results:**

Of 849 admissions, the mean age was 42.5 years and mean SAPS 3 score was 48.1. Comorbidities included hypertension (30.5%), HIV (32.6%), diabetes (13.3%), CKD (7.8%) and active tuberculosis (6.2%). The most common reason for admission was trauma (26%). AKI developed in 497 (58.5%). Male gender, illness severity, length of stay, vasopressor drugs and sepsis were independently associated with AKI. AKI was associated with a higher in-hospital mortality rate of 31.8% vs 7.23% in those without AKI. Age, active tuberculosis, higher SAPS 3 score, mechanical ventilation, vasopressor support and sepsis were associated with an increased adjusted odds ratio for death. HIV was not independently associated with AKI or hospital mortality. CKD developed in 14 of 110 (12.7%) patients with stage 3 AKI; none were dialysis-dependent.

**Conclusions:**

In this large prospective multidisciplinary ICU cohort of younger patients, AKI was common, often associated with trauma in addition to traditional risk factors and was associated with good functional renal recovery at 90 days in most survivors. Although the HIV prevalence was high and associated with higher mortality, this was related to the severity of illness and not to HIV status per se.

## Background

Acute Kidney Injury (AKI) is commonly encountered in the Intensive Care Unit (ICU) [1], but with a widely variable reported incidence due to non-standardization of its definition [2]. Regardless of the definition used, AKI is a well-recognized independent risk factor for mortality, is associated with substantial morbidity and is a current major cause for global concern [3–6]. Furthermore, AKI requiring dialysis is now recognized as a risk factor for end stage kidney disease in the long term [7, 8] and is associated with poor long-term quality of life after ICU discharge [9, 10].

AKI has been well characterized in high income (HI) countries and appears to be increasing in incidence [5, 11–13]. However, there is a marked paucity of data from African ICU’s concerning the incidence, aetiology and effect of AKI on mortality and functional renal recovery, where the prevalence of HIV and trauma is high and where resources are often limited [6, 14, 15]. Renal replacement therapy is an expensive [16] and scarce resource in South Africa [17] and in particular, in the rest of sub-Saharan Africa [18, 19].

The International Society of Nephrology has boldly called for 0by25: The elimination of preventable deaths from AKI by 2025 [20]. Timely diagnosis and prevention remain the most important strategies. Accordingly, understanding the epidemiology of AKI in lower and middle income (LMI) countries must be a key step in tackling this problem. Our aim therefore, was to describe the epidemiology of AKI in patients admitted to our South African multidisciplinary ICU, where there is currently a high prevalence of HIV and trauma-associated admissions. We sought to characterize the factors associated with the development of AKI, the effect of HIV on AKI as well as survival and to report on the 90-day renal function outcome of all who developed AKI.

## Methods

### Study design and setting

An observational prospective design was used. Cohorts were divided into those who did and did not develop AKI (prior to and/or after admission to the ICU) as well as by HIV status where known. All patients older than 12 years admitted to the Livingstone Hospital ICU between 3 January 2017 and 3 January 2018 were included. Patients who died within 6 h of being admitted to ICU, those who were brain-dead awaiting organ harvesting, and patients with known or presumed end stage kidney disease were excluded from the study.

The Livingstone Hospital adult ICU is a tertiary service, closed, multi-disciplinary 16-bed unit serving a catchment area of 1.6 million people. The hospital is government-funded and is located in the Nelson Mandela Bay Metropole in South Africa where 1.15 million people live in an urban setting, 12.3% of whom live in informal shack dwellings and 36.6% of whom are unemployed. The balance of 450,000 people live in surrounding rural areas within a radius of 250 km [21]. Full time consultant supervision is provided by two intensivists and two nephrologists. The provision of dialysis is also government-funded and not restricted within the ICU or by HIV status. However, general prognosis and current resource limitations are taken into consideration prior to the admission of any patient to the ICU [22]. The modality of dialysis is chosen by the treating consultant based on clinical status with a preference for intermittent haemodialysis or sustained low efficiency daily dialysis (SLEDD) due to cost constraints. Continuous renal replacement therapy (CRRT) is reserved for severely haemodynamically unstable patients and for those with raised intracranial pressure. Referring disciplines include medicine, trauma, general surgery, urology, neurosurgery, orthopaedics, obstetrics and gynaecology. Elective cardiology and cardio-thoracic surgery have their own dedicated ICU. Obstetrics also has their own high care although patients with advanced organ dysfunction are referred to our unit.

### Definition of acute kidney injury

AKI was diagnosed and staged according to the Kidney Diseases Improving Global Outcomes (KDIGO) definition [23]. A normal serum creatinine not older than 90 days was assumed to be the baseline where available, as recommended [24]. The cause of AKI was determined by the treating intensivist/nephrologist and more than one cause could be assigned.

### Definition of outcomes

AKI was recorded as resolved once creatinine improved to the known or presumed baseline. If renal function had not recovered by hospital discharge, patients were followed up for at least 90 days following ICU discharge or until renal recovery. Patients who had not recovered their renal function by this time were deemed to have chronic kidney disease (CKD).

### Ethical approval

Approval for the study (protocol number: 067/2016) was granted by the Walter Sisulu University Human Research Ethics Unit. Since we were conducting a non-experimental study that would not influence clinical decision-making or patient management, the need for study participant consent was waived by the Ethics Unit.

### Data collection and management

Demographic data including age, sex, race and details related to co-morbidities were recorded. For patients known with HIV, a premorbid CD4 count and viral load was recorded, where available. The Simplified Acute Physiology Score 3 (SAPS 3) [25] was calculated within the first hour of ICU admission. The Sequential Organ Failure Assessment (SOFA) [26] was calculated 24 h after admission and every third day thereafter, or sooner if the patient’s condition deteriorated. Vasopressor and mechanical ventilation requirements were also recorded. Cause of AKI, renal replacement modality and creatinine on admission, peak and discharge were recorded for patients in the AKI cohort. Sepsis was defined using Sepsis-3 criteria [27].

### Statistical analysis

Data were exported from the Research Electronic Data Capture (REDCap) hosted at the University of Cape Town [28] and analyzed with RStudio, 2017 (Version 3.4.2). Hypothesis tests were considered significant if the two-sided *p*-value < 0.05. Continuous data were tested for normality using the Kolmogorov-Smirnov, Shapiro-Wilk, Anderson-Darling and Pearson’s chi-squared tests. Normally distributed data are reported as means (standard deviation) and skewed data as medians (interquartile range). Discrete data are presented as numbers (percentages). The student’s t-test and the Mann-Whiteney U test were used to compare continuous data and the Chi-square and Fischer’s exact test were used for discrete data, as appropriate. Missing outcome data (*n* = 12) were analysed using multiple imputation. Hazard ratios for mortality by AKI and HIV status were calculated using the Cox proportional hazards model. Multivariate logistic-regression models were used to determine associations of developing AKI and dying. Variables by bivariate analysis with an alpha level < 0.1 between AKI and non-AKI cohorts as well as known predictors for AKI (age, sex, hypertension, active malignancy and admission SAPS 3 score) were included in the model. KDIGO stages 1, 2 and 3 were compared to patients who did not develop AKI as reference.

### Results

**Fig. 1:**
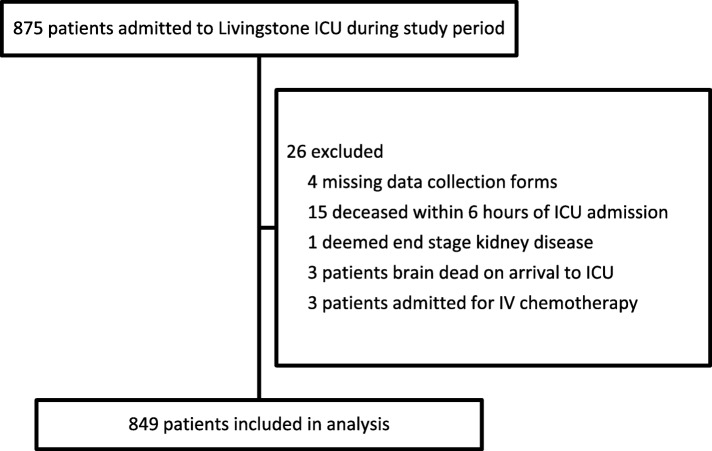
Diagram of patients included in the analysis

A total of 875 patients were admitted to the ICU during the study period and 26 were excluded from the analysis; Fig. 1 details the reasons for exclusion. Vital status after ICU discharge could not be established for 12 patients due to in-patient transfers to other hospitals and the unavailability of further records; six were in the AKI cohort, and outcomes were imputed.

In-hospital mortality was 21.6% while mortality in ICU was 13.4%. The most common diagnoses admitted were: Assault (14%), motor and pedestrian vehicle accidents (12%), acute abdomen (10%), pneumonia (6%; including cases later identified as tuberculosis) and self-inflicted drug/toxin overdose (3.5%). Admissions included surgical emergencies (*n* = 451, 53.1%), medical emergencies (*n* = 261, 30.7%), surgical elective cases (*n* = 110, 13%) and obstetric emergencies (*n* = 27, 3.2%). Table 1 shows the baseline characteristics of all patients admitted to the ICU and by AKI status.

**Table 1:** Baseline characteristics of all patients admitted to the ICU

Characteristic	All patients *n* = 849	AKI *n* = 497	No AKI *n* = 352	*p*-value
Demographics				
Mean age, years^a^ (SD)	42.5 (16.8)	43.7 (16.8)	40.9 (16.6)	0.017
Male gender^a^ %	58.9	61.2	55.7	0.110
Race^a^ %				
Black African	58.0	60.5	54.3	0.067
Mixed Ancestry	27.4	26.0	29.6	0.248
BCaucasian	13.8	12.7	15.3	0.267
Other	0.8	0.8	0.8	0.827
Co-morbidities				
Diabetes^a^ %	13.2	16.1	9.1	0.003
Mean HbA1c % (SD)	9.9 (3.2)	10.3 (3.1)	9.1 (3.3)	0.142
Hypertension^a^ %	31.6	32.2	30.7	0.641
Ischaemic heart disease %	4.2	4.6	3.7	0.506
Active Tuberculosis %	6.1	6.6	5.4	0.457
Chronic kidney disease^a^ %	7.7	6.4	9.4	0.113
Mean CKD eGFR (SD)	32 (19)	40 (16)	21 (1)	< 0.001
Epilepsy %	4.8	5.0	4.6	0.746
Malignancy^a^ %	3.3	3.6	2.8	0.530
HIV				
Known status, *n* = 472 (56.1%)				
*Positive, n (%)*	155 (32.6)	105 (35.1)	50 (28.3)	0.010
*Negative, n (%)*	321 (67.4)	194 (64.9)	127 (71.8)	0.382
In HIV positive cohort:				
Median CD4 count, cells/μL (IQR)	318(156–493)	205 (119–391)	404 (308–513)	0.025
Median viral load, log (IQR)	2.0 (1.98–4.04)	2.0 (1.88-3.62)	2.5 (2.00–4.64)	0.234
Receiving HAART^a^ %	11.9	14.5	8.2	0.006
Severity of illness				
Emergency admissions %	87.0	92.8	79.0	< 0.001
Mean SAPS 3 score^a^ (SD)	48.1	54.0	39.7	< 0.001
Median highest SOFA score^a^ (IQR)	4 (1–6)	6 (3–9)	2 (1–4)	< 0.001
Sepsis and septic shock^a^ %	30.2	43.1	11.9	< 0.001
Ventilated^a^ %	53.7	64.8	38.2	< 0.001
median ventilator days (IQR)	3 (1–7)	3 (1–8)	2 (1–4)	< 0.001
Required vasopressors^a^ %	25.0	38.8	5.4	< 0.001
ARDS^a^ %	4.4	6.8	0.9	< 0.001
Median ICU days (IQR)	3 (1–6)	4 (2–8)	2 (1–4)	< 0.001
ICU length of stay > 7 days^a^ %	24.7	33.2	12.8	< 0.001
Deceased in ICU %	13.0	20.5	2.3	< 0.001

Abbreviations: *AKI* Acute Kidney Injury, *CI* Confidence interval, *SD* Standard deviation, *IQR* Interquartile range, *CKD* Chronic Kidney Disease, *eGFR* Estimated Glomerular Filtration Rate, *HAART* Highly Active Antiretroviral Therapy, *SAPS 3* Simplified Acute Physiology Score 3, *SOFA* Sequential Organ Failure Assessment, *ARDS* Acute Respiratory Distress Syndrome. ^a^Variables included in multivariate analysis

### Severity and causes of AKI

AKI developed in 497 patients (58.5%), 80.5% of whom were diagnosed with AKI on admission to the ICU. The maximum KDIGO stage was stage 1 in 138 (27.7%), stage 2 in 152 (30.6%) and stage 3 in 207 (41.7%) patients respectively.

**Fig. 2:**
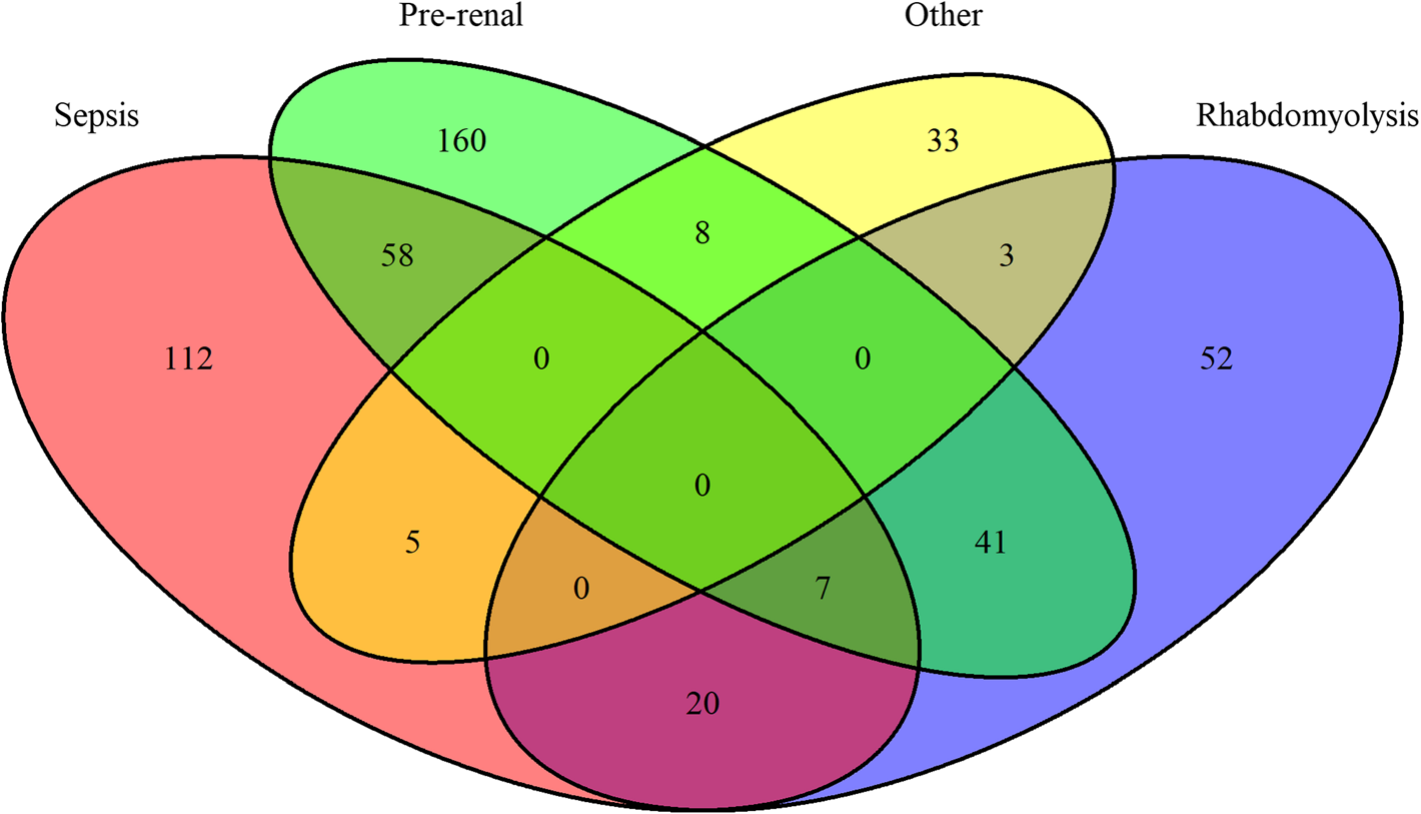
Identifiable causes of AKI. A pre-renal aetiology (such as hypovolaemic shock) was identified in 55.1%, sepsis in 40.6% and rhabdomyolysis, usually related to trauma, in 24.8%. “Other” included obstructive uropathy, direct drug nephrotoxicity, hypertensive crisis, thrombotic microangiopathy, and rapidly progressive glomerulonephritis. More than one cause may have been implicated in the same patient, especially if the patient developed more than one episode of AKI during the same admission

Figure 2 shows the main causes identified for developing AKI. Herbal ingestion was only documented in 4 patients and 12 patients were exposed to anti-tuberculous drugs (rifampicin and isoniazid). Of the 105 HIV positive patients who developed AKI, 24 had received tenofovir prior to AKI diagnosis. Histology was obtained in situations where the cause of AKI was not clear or the clinical course of the patient was unclear. Kidney biopsies were performed in 5 patients (1% of the AKI cohort), only 1 of whom had HIV which showed interstitial HIV associated nephropathy. The others were in HIV negative subjects; 3 showed features of mesangiocapillary glomerulonephritis, 1 of which was crescentic and 1 had features of ascending pyelonephritis.

### Risk factors associated with AKI

Risk factors associated with AKI are presented in Table 2. On multivariate analysis, male gender, increased SOFA and/or SAPS 3 score, increased length of stay, the need for vasopressor drugs and the development of sepsis or septic shock were independently associated with the development of AKI by logistic-regression modeling. HIV was not independently associated with AKI.

**Table 2:** Crude and Adjusted odds ratios determined by multivariate analysis exploring risk factors associated with AKI

	Crude odds ratio (95%CI)	Adjusted odds ratio (95%CI)	*p*-value
Age, per 10 year increase	1.10 (1.002; 1.018)	1.11 (0.999; 1.260)	0.079
Male gender	1.22 (0.92; 1.608)	1.44 (1.008; 2.058)	0.045
Diabetes	1.88 (1.228; 2.945)	1.69 (0.982; 2.947)	0.059
Baseline CKD	0.64 (0.382; 1.066)	0.32 (0.163; 0.634)	0.001
Receiving HAART	1.95 (1.245; 3.137)	1.64 (0.940; 2.920)	0.086
Admission SOFA score	1.48 (1.391; 1.585)	1.33 (1.215; 1.456)	< 0.001
SAPS 3 score	1.07 (1.062; 1.088)	1.03 (1.013; 1.047)	< 0.001
ICU length of stay, days	1.12 (1.081; 1.155)	1.04 (1.007; 1.072)	0.026
Required mechanical ventilation	3.05 (2.299; 4.072)	0.56 (0.359; 0.860)	0.003
Required vasopressors	11.15 (6.954; 18.878)	2.52 (1.392; 4.700)	0.003
Developed ARDS	12.39 (3.734; 76.752)	3.20 (0.840; 21.071)	0.137
Developed sepsis or septic shock	5.50 (3.839; 8.038)	1.81 (1.119; 2.929)	0.016
HIV Infection^a^	1.42 (0.952; 2.15)	0.60 (0.272; 1.305)	0.198

Abbreviations: *HAART* Highly Active Antiretroviral Therapy, *SOFA* Sequential Organ Failure Assessment, *SAPS 3* Simplified Acute Physiology Score 3, *ARDS* Acute Respiratory Distress Syndrome. ^a^ Analysis restricted to only those with a known HIV serostatus (*n* = 472)

### Use of renal replacement

**Fig. 3:**
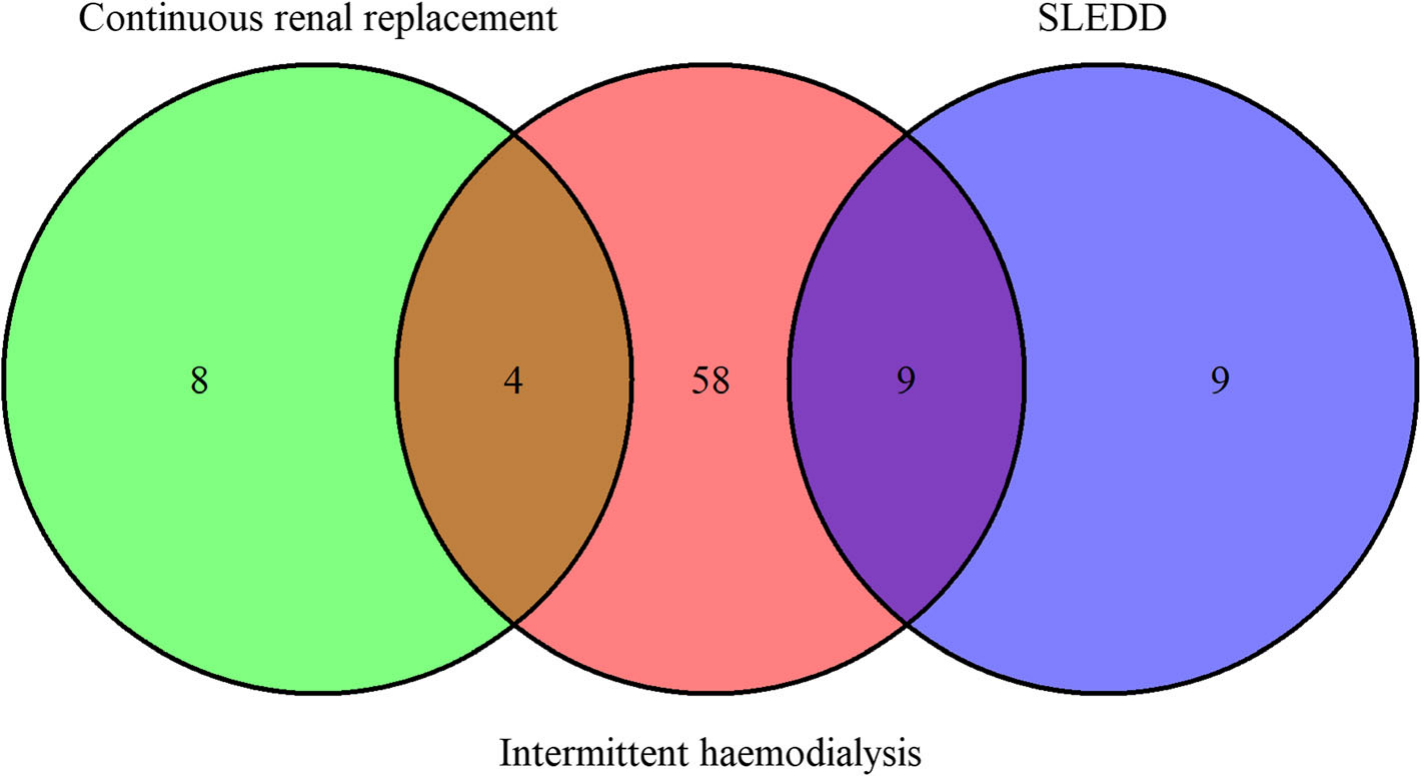
The relationship between modes of renal replacement that were used Abbreviations: CRRT, Continuous Renal Replacement Therapy; IHD, Intermittent Haemodialysis; SLEDD, Sustained Low Efficiency Daily Dialysis.

Eighty-eight patients were dialyzed (42.5% of patients with stage 3 AKI, 18.0% of the cohort with AKI and 10.4% of the entire cohort), 67.0% of whom were initiated within 24 h of arrival to ICU. Intermittent haemodialysis was used in most (80.7%; Fig. 3). The most common indications for dialysis initiation were life-threatening hyperkalaemia (44.3%), uraemic symptoms such as encephalopathy or seizures (34.1%) and refractory metabolic acidosis (34.1%).

### Mortality and AKI

**Fig. 4:**
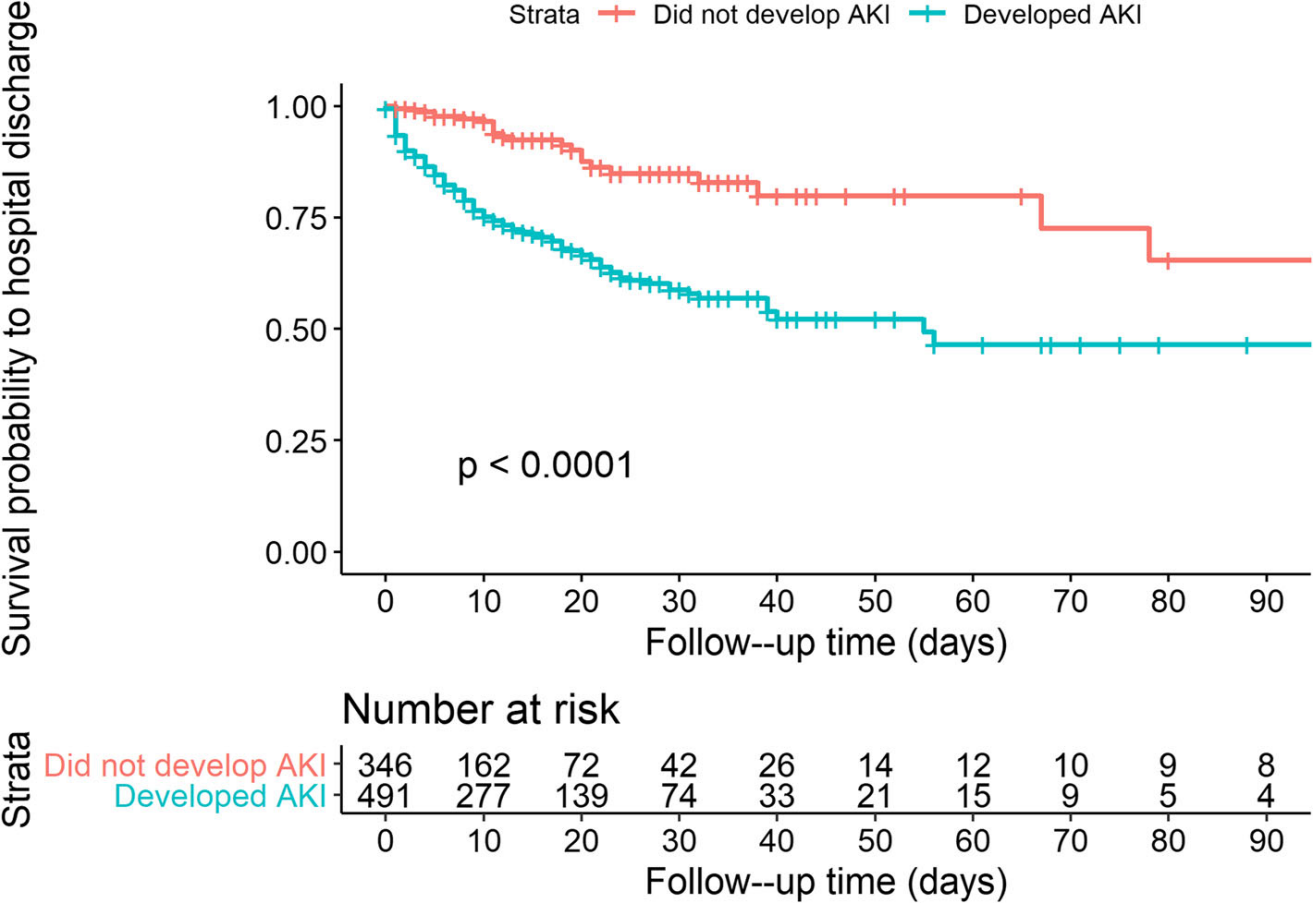
Kaplan Meier plot showing survival probability for patients following ICU admission who developed AKI and those that did not

**Fig. 5:**
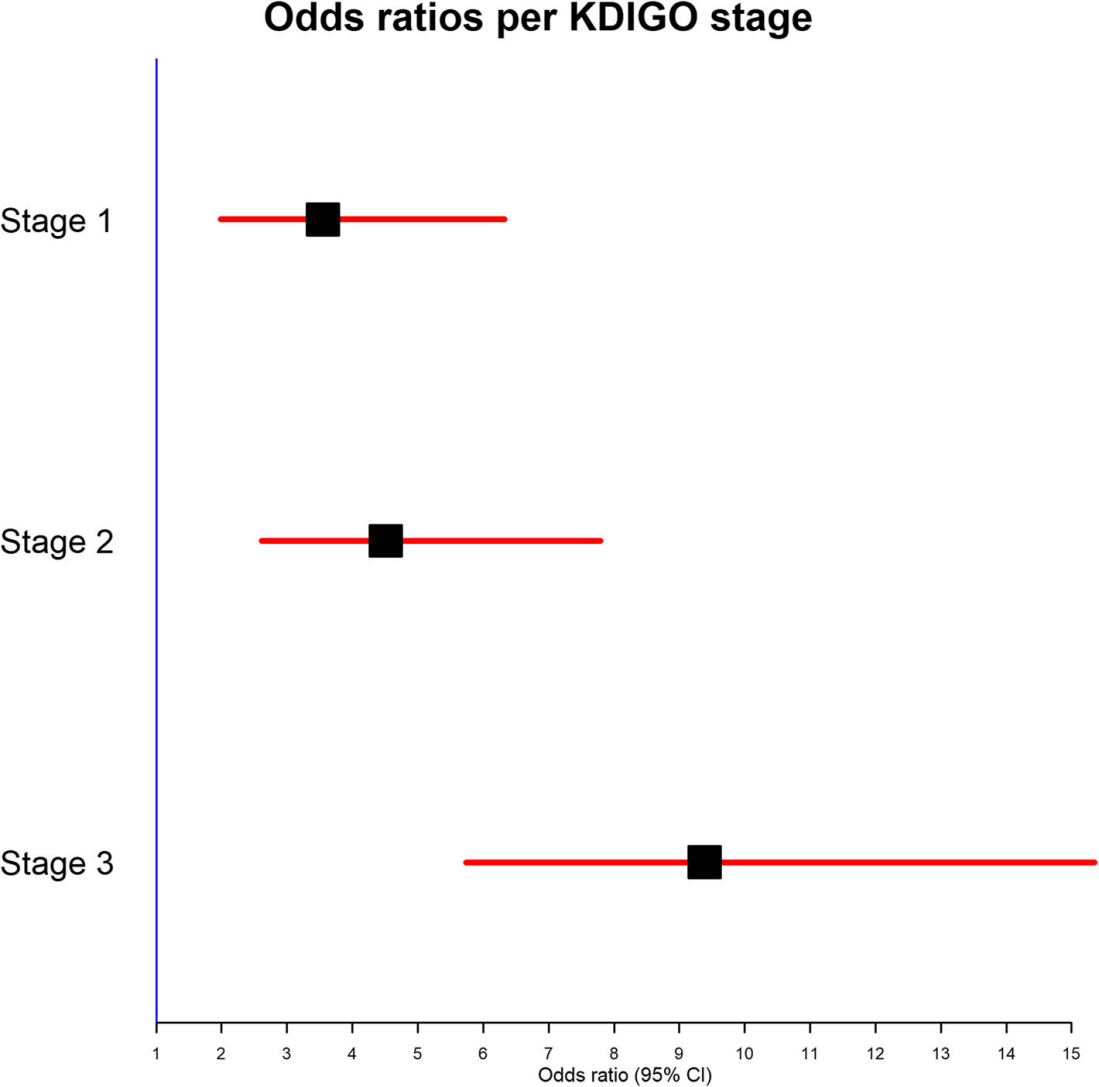
The odds of dying for increasing KDIGO stage of severity

The development of AKI was associated with a higher in-hospital mortality rate of 31.8% compared to 7.2% in those without AKI (Hazards Ratio 4.07, 95% CI 2.66; 6.21; logrank *p* < 0.001 (Fig. 4)). Further, the odds of dying increased stepwise with increasing KDIGO AKI stage (Fig. 5). The absolute in-hospital mortality rate was 29/134 (21.6%), 39/150 (26.0%) and 87/206 (42.2%) in stage 1, 2 and 3 AKI respectively and 32/88 (36.4%) for those who received dialysis. In those receiving exclusively intermittent haemodialysis, mortality was 31.0% compared to 55.5 and 62.5% for those who received exclusively SLEDD or CRRT, respectively [adjusted OR (95%CI) for CRRT: 6.59 (1.55; 27.88); *p*-value 0.01].

In the AKI cohort, an increased adjusted odds ratio for death was observed with increasing age, active tuberculosis, higher SAPS score, receipt of mechanical ventilation, receipt of vasopressor support and in those with sepsis (Table 3).

**Table 3:** Multivariate analysis for predictors of mortality in the AKI cohort (*n* = 497)

Covariate	Crude odds ratio (95% CI)	Adjusted odds ratio (95% CI)	*p*–value
Age, per 10 year increase	1.16 (1.030; 1.300)	1.33 (1.12; 1.59)	0.002
Gender (male)	0.88 (0.596; 1.296)	0.96 (0.584; 1.588)	0.878
Diabetes	0.8 (0.462; 1.348)	0.47 (0.223; 0.963)	0.043
Active malignancy	0.82 (0.259; 2.218)	1.03 (0.25; 3.782)	0.963
Active tuberculosis	2.14 (1.042; 4.370)	2.84 (1.14; 7.079)	0.025
CKD	0.87 (0.372; 1.880)	1.12 (0.357; 3.331)	0.840
Hypertension	1.1 (0.733; 1.665)	1.47 (0.790; 2.81)	0.224
Receiving HAART	0.99 (0.585; 1.719)	1.37 (0.670; 2.88)	0.398
SAPS 3 score	1.07 (1.054; 1.089)	1.04 (1.026; 1.065)	< 0.001
ICU days	0.99 (0.971; 1.010)	0.96 (0.929; 0.985)	0.004
Mechanical ventilation	5.00 (3.087; 8.420)	2.08 (1.084; 4.062)	0.029
Requiring vasopressors	7.26 (4.782; 11.182)	3.99 (2.318; 6.961)	< 0.001
ARDS	1.43 (0.678; 2.930	0.82 (0.335; 1.973)	0.661
Sepsis	3.15 (2.127; 4.686)	1.8 (1.047; 3.083)	0.033
HIV status^a^	1.76 (1.05; 2.94)	1.11 (0.56; 2.15)	0.769

^a^Analysis restricted to only those with AKI and a known HIV status (*n* = 296). See survival analysis for HIV-AKI cohort in Fig. 6.

Abbreviations: *HAART* Highly Active Antiretroviral Therapy, *SAPS 3* Simplified Acute Physiology Score 3, *ARDS* Acute Respiratory Distress Syndrome

**Fig. 6:**
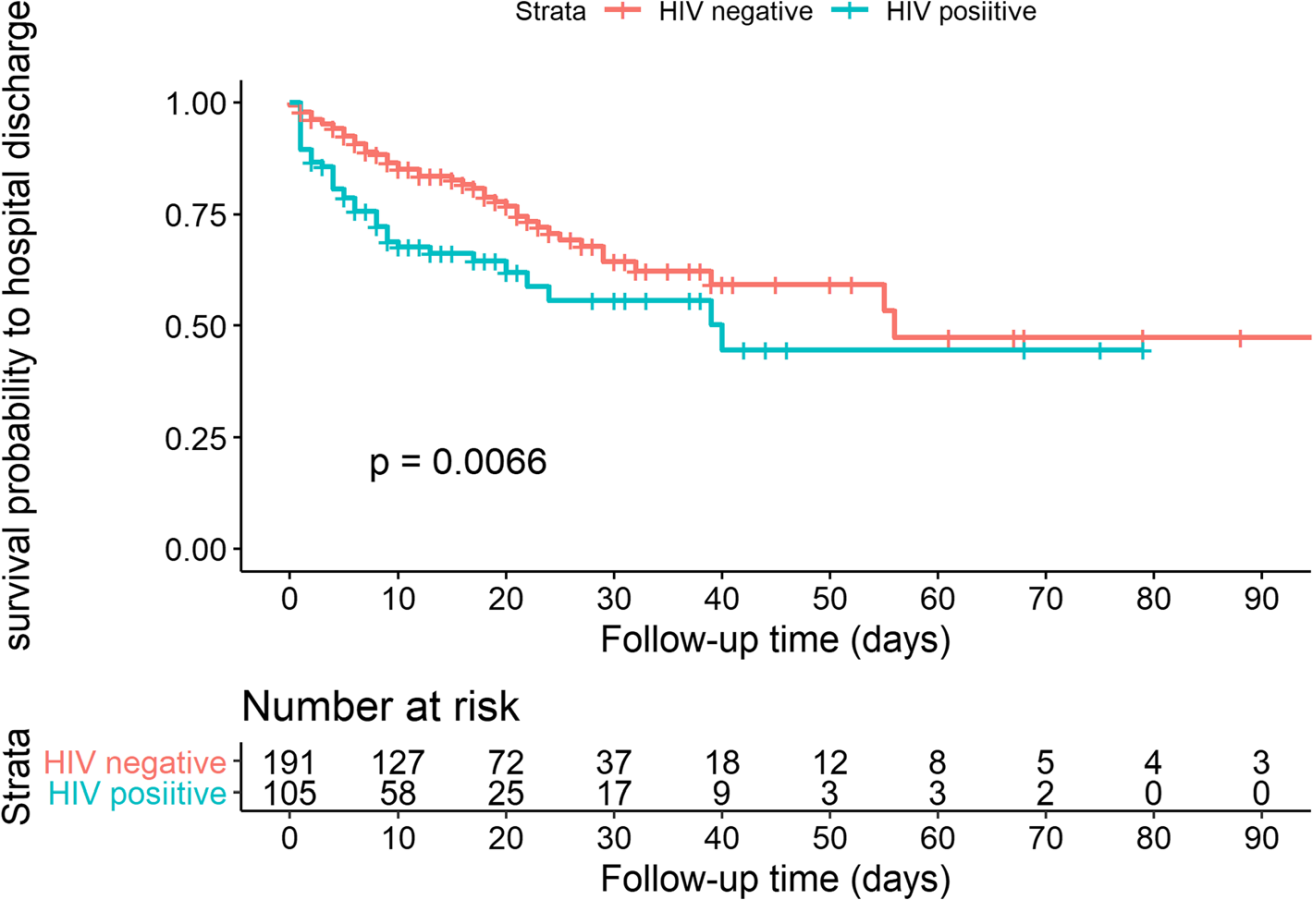
Kaplan Meier plot showing the association between survival probability by HIV status for those with AKI, Hazard ratio (95%CI) = 1.8 (1.18; 2.74). The analysis was restricted to only those with AKI and a known HIV status (*n* = 296)

HIV infection was associated with worse survival in those with AKI (Fig. 6). However, on multivariate analysis, HIV was not found to be an independent risk factor for mortality (Table 3). Patients with HIV and AKI were more severely ill on admission with mean SAPS 3 score (SD) of 58.2 (15.9) versus 53.3 (13.8), *p* = 0.007 and SOFA (IQR) of 8 (4; 11) versus 6 (3; 9), *p* = 0.01. They also had a higher likelihood of having active tuberculosis [OR (95%CI) = 3.55 (1.42; 9.36), *p* = 0.008]. The median premorbid CD4 count was significantly lower in those with AKI than without (204.5 cells/microliter versus 404 cells/microliter, *p* = 0.025) but viral suppression was similar in both groups (viral load log 2.0 vs 2.5 respectively, *p* = 0.234). Although ICU length of stay was less in HIV patients with AKI (median ICU days 3 vs 6, *p* = 0.002), the proportion of patients with HIV who died within the first 24 h was higher at 10.5% compared to 2.6% in those who were HIV negative, *p* = 0.009.

### Renal recovery from AKI

Functional renal recovery was assessed at 90 days after hospital discharge in patients with AKI who survived (*n* = 332): Full recovery occurred in 236 (100%) of those who developed maximum KDIGO stage 1 or 2 AKI and 96 of 110 (87.3%) who developed stage 3 AKI. CKD was therefore established in 14 patients (12.7% of survivors) with a median creatinine (IQR) of 175 (137; 424) μmol/L. Non-recovery of renal function in survivors was associated with a higher admission creatinine (670 vs 257 μmol/L; *p*-value 0.004) and receiving dialysis (64.3% vs 32.3%, *p* = 0.043), but not with admission severity score (SAPS 3 admission score = 55.1 versus 56.1, *p* = 0.811), premorbid creatinine values (76 μmol/L; IQR 60; 228 versus 76umol/L; IQR 56; 130, *p* = 0.796) nor receiving prolonged dialysis modalities like SLEDD and CRRT (44.4% versus 29.03% respectively, *p* = 0.667). The group that did not recover was less likely to have pre-renal failure (14.3% versus 51.0%, *p* = 0.022) as a cause for AKI. This group also tended to have more diabetes (35.7% vs 15.6%, *p* = 0.069) and hypertension (57.1% vs 31.3%, *p* = 0.057), a lower CD4 count if HIV positive (55, IQR 45; 65, vs 165, IQR 131; 312; *p* = 0.067) and a higher HIV viral load (log 4.06 vs log 1.94, *p* = 0.247). HIV infection itself and the receipt of HAART was not associated with non-recovery of renal function in survivors, *p* = 0.972 and 0.886 respectively. No patients were dialysis dependent at 90-days.

### Other differences between HIV positive and negative patients with AKI

HIV positive patients with AKI were more likely to be of Black African ethnicity (85.7% vs 59.2%, *p* < 0.001), to be an emergency admission (92.4% vs 23.0%, p < 0.001), to have a medical rather than surgical problem (73.3% vs 26.2%, p < 0.001), to have sepsis (41.3% vs 32.1%, *p* = 0.049) and to have active tuberculosis (15.5% vs 6.85%, *p* < 0.003).

## Discussion

This is the largest prospective African study of AKI in critically ill adults with an HIV seropositive rate of 32.6%. Similar studies include reports from Morocco (*n* = 97) [29], the Democratic Republic of Congo (DRC, *n* = 476) [30] and Egypt (*n* = 532) [31]. However, only the DRC study reported HIV prevalence, which was low at 2.9%. Consistent with other LMI countries, patients were younger than in HI country cohorts (mean age 40.6 years) with lower comorbidity rates [1, 18, 19, 29–32], mostly of Black African ethnicity, and were representative of the local community that we serve [21].

As in other African [29–31] and HI country [1] ICU-based studies, AKI was very common in our cohort and affected nearly two thirds (58.5%) of all patients admitted to the ICU. AKI was associated with male gender, higher severity of illness, more sepsis, longer ICU stay and the need for vasopressors. Pre-existing CKD was negatively associated with AKI but reflects an admission bias against very ill patients with CKD to the unit due to a lack of resources to continue with chronic renal replacement therapy in most. Increased age, severity of illness, sepsis, mechanical ventilation, the use of vasopressors and the presence of tuberculosis were independently associated with mortality in those with AKI. Furthermore, increasing stage of AKI showed a stepwise increase in the risk of mortality.

Although our unit admits emergency medical as well as elective surgical cases, just over a quarter of all admissions were trauma related, reflecting the alarmingly high levels of interpersonal violence and road traffic accidents prevalent in South Africa. In 2010, interpersonal violence and road injury combined were the second leading cause of death and disability adjusted life years lost in South Africa, after HIV which was the leading cause [33]. Consequently, a large proportion of AKI was attributable to hypovolaemic shock (55.1%) and rhabdomyolysis (24.8%) in keeping with the degree of trauma-related admissions. Two recent retrospective studies in South Africa also highlighted AKI in trauma victims as a major contributor to morbidity and mortality [34, 35]. This reflects a major change in the epidemiology of AKI in South Africa where older reports have not highlighted trauma as a major cause of AKI [14, 15, 36] and has important public health implications for health administrators. Sepsis was also a common precipitant of AKI at 40.6% which is similar to that reported in other LMI country studies in the critically ill [1, 31, 37]. Herbal and traditional medicine use is not a prominent cause of AKI in our region compared with prior reports from other regions [38, 39]. Tropical diseases such as malaria are not endemic in our region and are therefore also not common precipitants of AKI.

Severe AKI (KDIGO stage 3) was common, affecting 41.4% of all those with AKI and 24.4% of all admissions. The number of patients dialyzed (10.4% of all admissions) was slightly lower than in the large Acute Kidney Injury–Epidemiologic Prospective Investigation (AKI-EPI) study where 13.5% of all admissions were dialyzed [1] and may be explained by local practice to usually delay the initiation of dialysis until more classic indications exist. Notwithstanding this fact, most patients requiring dialysis were initiated early (within 24 h of ICU admission) reflecting the advanced state of organ dysfunction at admission and late presentation that is common in LMI countries [32]. Although the AKI-EPI study was multinational, only 34 patients from Africa were included. Continuous renal replacement therapies are available in our center but cost in excess of 10 times more than intermittent dialysis. As such, CRRT is reserved for specific indications and the rate of CRRT use was much lower in our study at 13.6% compared to 75.2% in the AKI-EPI study. Of those who developed AKI and survived, a significant proportion of patients with stage 3 AKI did not recover renal function fully (12.7%).

South Africa has a very high HIV burden of 7.9 million people living with HIV as well as the largest number of people on antiretroviral therapy in the world [40]. The HIV seropositive rate of 32.6% in our cohort is consistent with the known background prevalence of HIV in our province of 25.2% in adults aged 15–49 when measured in 2018 [40]. This is vastly different to a retrospective study in a South African medical ICU in 2004 [41] where only three patients (6.5%) in the AKI cohort were HIV positive. In our study, active tuberculosis was very common, affecting 1 in 16 of all admissions. This is likely in part due to the high prevalence of HIV, but also due to the high reported background incidence of tuberculosis in the community of 1095 cases/100 000 per year [42]. Whilst tuberculosis was not associated with AKI per se, it was independently associated with mortality. Many cases of active tuberculosis were diagnosed during ICU admission through microbiological means and many were not the primary cause of admission.

HIV infection was associated with higher mortality, as well as the presence of sepsis and active tuberculosis. It was also associated with increased severity of illness and the need for emergency admission. Length of stay was shorter for those with HIV, but reflects earlier mortality in those with increased severity of illness. As in other studies in the post-HAART era [36, 37, 43–45], it would appear from our data that traditional predictors of mortality such as higher severity of illness are implicated in predicting mortality and not HIV status, CD4 count, viral suppression nor the use of HAART.

Proportionally few (22.9%) HIV positive patients that developed AKI were receiving Tenofovir-based HAART at the time of ICU admission. Although there was little difference in viral suppression between groups, the CD4 count was significantly lower in the group that developed AKI thereby placing patients at risk for the immune reconstitution inflammatory syndrome (IRIS) [46]. While tenofovir has been shown to be nephrotoxic [47], we hypothesize that the pathogenesis of AKI in at least some of our HIV patients was related to the often recent initiation of HAART with the development of an unmasking tuberculosis-associated IRIS [48] and consequent tuberculosis sepsis syndrome with associated AKI. This has previously been reported [49] and is likely to be under-recognized.

### Study limitations and strengths

This study needs to be viewed in the context of its limitations. We may have underestimated the incidence of AKI since we were unable to reliably utilize the KDIGO urine output criterion for diagnosis as patients admitted to the ICU were not always weighed or catheterised. Secondly, 43.9% of all patients admitted to the ICU were not tested for HIV. All patients who were able to consent were encouraged to have an HIV test; however, patients that were moribund or confused were not tested for HIV without indication or consent. On the other hand, to our knowledge, this is the largest published prospective cohort of critically ill adults with HIV and AKI [50]. The study was inclusive of all major disciplines with the exception of cardiothoracics and the loss to follow up was low at 1.4%. Standardised criteria for the diagnosis of all stages of AKI were used and 90-day renal recovery data was obtained.

## Conclusion

In this large prospective multidisciplinary ICU cohort of younger patients in a LMI country with a high HIV prevalence and many trauma related admissions, AKI was frequently encountered, and was associated with a high mortality, but good functional renal recovery in most survivors. While HIV infection was associated with higher mortality, this was due to increased severity of illness, not HIV status per se.

## Data Availability

Datasets are accessible from the Mendeley Data public repository available at 10.17632/7f6yxz2d4c.1

## Abbreviations

AKI: Acute Kidney Injury
ARDS: Acute Respiratory Distress Syndrome
CD4: Cluster Differentiation 4
CKD: Chronic Kidney Disease
CRRT: Continuous Renal Replacement Therapy
eGFR: Estimated Glomerular Filtration Rate
HAART: Highly Active Antiretroviral Therapy
HI: High Income
HIV: Human Immunodeficiency Virus
ICU: Intensive Care Unit
IHD: Intermittent Haemodialysis
IQR: Interquartile Range
IRIS: Immune Reconstitution Inflammatory Syndrome
IV: Intravenous
KDIGO: Kidney Diseases Improving Global Outcomes
LMI: Low-middle Income
REDCap: Research Electronic Data Capture
SAPS: Simplified Acute Physiology Score
SD: Standard Deviation
SLEDD: Sustained Low Efficiency Daily Dialysis
SOFA: Sequential Organ Failure Assessment
